# Eosinophil counts predict 3-year risk of major adverse cardiovascular and cerebrovascular events in carotid artery stenosis patients

**DOI:** 10.1038/s41598-025-06350-w

**Published:** 2025-07-01

**Authors:** Ruijing Zhang, Runze Chang, Xiaotong Qi, Xing Cheng, Lin Zheng, Chuanlong Lu, Maolin Qiao, Siqi Gao, Yaling Li, Jinshan Chen, Jie Hu, Honglin Dong

**Affiliations:** 1 Department of Vascular Surgery, The Second Hospital of Shanxi Medical University, 382 Wuyi Street, Taiyuan, Shanxi China; 2 Department of Nephrology, The Second Hospital of Shanxi Medical University, Taiyuan, China; 3 Institute of Vascular Disease, Shanxi Medical University, Taiyuan, Shanxi China

**Keywords:** Carotid artery stenosis, Major adverse cardiovascular and cerebrovascular events, Eosinophil counts, Prognosis, prediction model, Biomarkers, Cardiovascular diseases

## Abstract

Eosinophils are a part of the human immune system and have traditionally been thought to be associated with allergic reactions. Recent research has revealed their potentially important role in cardiovascular disease. We retrospectively collected data on 1155 patients who were diagnosed with carotid artery stenosis at our center from January 2016 to June 2021. These data included demographic information, laboratory measurements, and carotid ultrasounds. We constructed predictive models through binary logistic regression. A total of 1,155 patients were ultimately included in this study, among whom 157 experienced major adverse cardiovascular and cerebrovascular events (MACCE) within a three-year follow-up period. Multifactorial adjustment showed that previous operation history (OR 1.66[1.14–2.41], *P* = 0.008), previous history of stroke (OR 1.59[1.10–2.31], *P* = 0.014), total proteins (OR 0.97[0.95–0.99], *P* = 0.02), erythrocyte sedimentation rate(OR 1.01[1.01–1.02], *P* = 0.031), eosinophil counts(OR 4.62[1.60–13.35], *P* = 0.005) and treated with calcium channel blockers(CCBs) + β-blockers(BBs) (OR 3.08[2.04–4.63], *P* < 0.001) were independently associated with 3-year MACCE. Based on these key factors, the prediction model we constructed demonstrated satisfactory prediction performance, with an initial value of 0.739 for the area under the curve, which was stabilised at 0.721 after adjustment. After a dichotomous analysis of eosinophils based on cut-off values, we assessed their role in different outcomes. The results of the analysis showed that eosinophil counts was a significant risk factor for all the outcomes considered. Patients with carotid artery stenosis exhibiting a baseline eosinophil counts of 0.185*10^9^/L had a higher incidence of MACCE. Furthermore, a previous 3-year operation history, previous history of stroke, total proteins, erythrocyte sedimentation rate and treated with a combination of CCBs and BBs were also significantly associated with the occurrence of MACCE.

**Supplementary Information:**

The online version contains supplementary material available at 10.1038/s41598-025-06350-w.

## Introduction

Atherosclerotic cardiovascular disease (ASCVD) is the most prevalent disease worldwide, and its complications remain the leading cause of death globally^1,2^. The carotid artery is the main blood vessel supplying blood to the human brain, and major adverse cardiovascular events such as stroke caused by carotid artery lesions have become a serious public health concern^3,4^. The primary cause of carotid artery stenosis is the formation and progressive enlargement of atherosclerotic plaques, leading to luminal narrowing. With advancing age, both the incidence and severity of carotid artery stenosis increase significantly, thereby markedly elevating the risk of major cardiovascular events^5^. Aging, diabetes, hyperlipidemia, and hypertension are traditional risk factors for atherosclerosis. They contribute to the initiation and progression of atherosclerosis through mechanisms such as promoting lipid deposition, inducing low-grade chronic inflammation, triggering oxidative stress, and impairing endothelial function^6–10^. However, traditional ASCVD risk factors may not be reliable enough due to variations in problematic vessels, individual differences, and lifestyle factors. Therefore, it has become a trend to explore novel biomarkers for complete cardiovascular risk stratification to guide clinical prognostic interventions.

Eosinophils (EO) were first discovered by Paul Ehrlich^11^ in 1879. Eosinophils are members of the innate immune system. After maturation in the bone marrow, eosinophils enter the peripheral blood circulation, where they participate in the immune response. In the blood of healthy individuals, eosinophils make up 1–5% of the total number of leukocytes^12^. Although they are well known for their role in allergic reactions, eosinophils also regulate the immune balance by secreting a variety of inflammatory mediators and chemokines, which also play a crucial role in the prevention and treatment of non-allergic diseases^12–14^. More and more studies have clarified that it is significantly related to the occurrence and development of cardiovascular diseases^15–17^. Research has demonstrated that eosinophils can post-myocardial infarction preserve cardiac function and delay the development of an AngII-induced abdominal aortic aneurysm^18,19^. However, in atherosclerosis, eosinophils contribute to atherosclerosis by infiltrating into the vessel wall through the release of eosinophil cationic proteins (ECP) and by interacting with platelets^16,20^. In a large-scale clinical study from the UK Biobank, a significant association was found between eosinophil counts and cardiovascular disease and its mortality^21^. However, another study found lower eosinophil counts in patients with ST-segment elevation myocardial infarction(STEMI) who experienced major adverse cardiovascular events(MACE)^22^. In response to this seemingly contrary discovery, Norbert Gerdes^23^ shared his discovery that a high eosinophil counts in patients with stable cardiovascular diseases (CVD) predicted risk for MACE in the future. In contrast, acute injury may recruit eosinophils from the circulation due to injured tissue, which would result in a decrease in the number of eosinophils in the blood^23^. This hypothesis highlights that eosinophils may play different roles at different stages of the disease, thereby affecting their number in the circulation. However, the evidence is limited in regard to the occurrence of major adverse cardiac and cerebrovascular events (MACCE) after carotid stenosis. Therefore, the objective of this study was to determine whether the levels of eosinophil counts at admission could indicate the long-term prognosis of patients with carotid stenosis.

## Materials and methods

### Patient selection

This study followed the Declaration of Helsinki, was approved by the Institutional Ethics Committee of the Second Hospital of Shanxi Medical University, and the informed consent form were obtained. (No. 2023126) We retrospectively recruited 1892 patients who underwent carotid ultrasound between January 2016 and June 2021, of whom 1660 were diagnosed with carotid stenosis. Carotid stenosis was considered to be more than 30% stenosis according to the criteria of the North American Symptomatic Carotid Endarterectomy Trial (NASCET) group^24^. According to the Carotid artery stenosis: gray-scale and Doppler US diagnosis—Society of Radiologists in Ultrasound Consensus Conference, carotid occlusion was considered to be either no blood flow in the lumen or failure to detect lumen^25^. Symptomatic patients presented with transient ischemic attack, transient blackout, or minor nondisabling stroke within 180 days prior. Conversely, asymptomatic patients^26^.

**Fig. 1:**
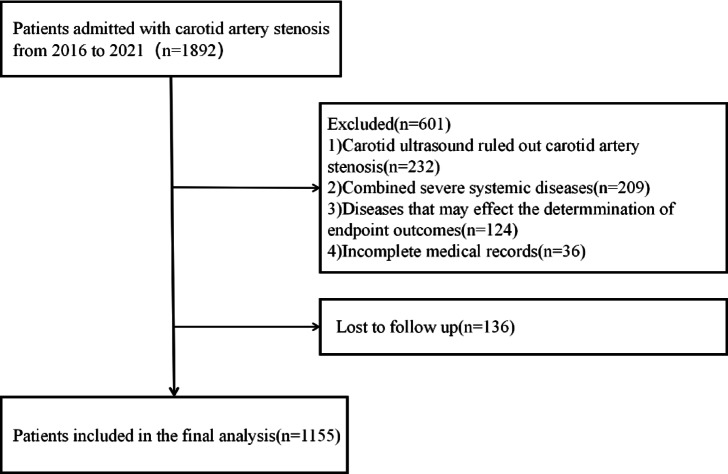
Flow diagram of patient selection.

Patients with acute infections, severe renal disease (serum creatinine > 1.4 mg/dL), severe hepatic disease (hepatic function parameters > 3 × upper limit of normal), hematologic disorders, autoimmune disorders, malignant neoplasms, or incomplete critical medical records were excluded. In addition, preexisting events that could have influenced the determination of the endpoint outcome of the present study, such as: within the past 30 days myocardial infarction or severe stroke, were also excluded as confounders. A total of 1291 patients were enrolled in this study. Three investigators without prior knowledge of the patients’ clinical data followed up all patients by telephone to determine whether MACCE had occurred. Ultimately, we successfully followed up on 1155 individuals (Fig. 1).

### Data collection

All data were collected from medical electronic records by clinicians who were unaware of the purpose of the study. This included patients’ demographic characteristics [Age, Gender, Height, Weight, and body mass index (BMI)], cardiovascular risk factors [Smoke, Drunk, Hypertension, Diabetes, history of stroke], laboratory parameters [Fibrinogen, Activated partial thromboplastin time, ALT, AST, Bilirubin, Creatinine, Urea nitrogen, Homocysteine, Lipids, Red blood cells, Leukocytes, Platelets, Hemoglobin, etc.], surgical history [CEA\CAS and surgery], medication [antiplatelet agents, hypoglycemic, beta-blockers, angiotensin-converting enzyme inhibitors (ACEIs)/angiotensin receptor blockers (ARBs), calcium channel blockers, etc.], and carotid ultrasound [carotid artery branch plaques, carotid branch of the largest plaque, maximum plaque length, maximum plaque width, maximum plaque area, etc.] Peripheral blood samples were collected after overnight fasting and blood biochemistry levels were determined in a central laboratory.

### Carotid ultrasound

Carotid ultrasound was performed by senior sonographers, who were not informed of the participants’ basic information and laboratory indicators in advance. All procedures are performed according to diagnostic criteria established by the American College of Radiology in 2003^25^. Participants were placed in a supine position and examined using an iU22 ultrasound system (Philips Healthcare) and a 6–10 array mhz linear array transducer. Bilateral common carotid artery (CCAs), internal carotid artery (ICAs), external carotid artery (ECAs), subclavian artery and vertebral artery diameter and stenosis rate were recorded accurately.

### Endpoints

The primary endpoint outcome of this study was the occurrence of MACCE, defined as a composite of transient ischemic attack(TIA), ischemic stroke, acute myocardial infarction(AMI), or all-cause death^27^. Deaths from clear causes of non-cardiovascular disease were excluded. Secondary endpoints included TIA\ischemic stroke, AMI. The outcome diseases were diagnosed by senior specialists or supported by examination reports.

### Missing value

Variables with missing values > 30% were excluded, and the remaining missing values were imputed through the ‘mice’ package in R. The imputation dataset was 20 times, and the with and pool function selected the imputation set that was most consistent with the original dataset.

### Statistical analysis

A retrospective cohort study was conducted, and statistical analysis was performed using R (version 4.2.2). Quantitative variables are expressed as the mean ± standard deviation (mean ± SD), while categorical variables are expressed as percentages (%). The Kolmogorov‒Smirnov test was used to check for a normal distribution. Student’s t test or X^2^ test was deployed to compare the variables of interest between the MACCE outcome groups. Receiver operating characteristic (ROC) curve analysis was carried out on significant continuous variables to determine the cutoff value with the highest sum of sensitivity and specificity in the predicted event. In light of the scientific objectives of this study, the statistical properties of the predictors, and the nature of the dataset, logistic regression was employed to systematically investigate the associations between risk factors and the outcome variable. Factors with significant univariate relationships with MACCE (*p* < 0.05) were included in the binary logistic multiple regression model. The reliability of the model was assessed using ROC curves, and the consistency of the model was confirmed using calibration curves.

## Results

A total of 1155 participants (68.37% male) were included. Traditional cardiovascular disease risk factors, including diabetes (28.21%), hypertension (61.03%), operation history (37.13%) and previous stroke history (30.92%), exhibited a high prevalence in this study population. It is noteworthy that 29.03% of the patients had symptomatic carotid artery stenosis (Table 1). The use of multiple imputation for missing variables did not significantly affect the results (Supplementary Table 1).

**Table 1:** Clinical characteristics compared between subjects With and Without 3-Year MACCE.

	ALL (N = 1155)	No MACCE	MACCE	*p* value
		N = 998	N = 157	
Age, y	65.08 (10.41)	65.09 (10.42)	65.01 (10.35)	0.928
Gender, %				0.983
Man	790 (68.40%)	682 (68.34%)	108 (68.79%)	
Woman	365 (31.60%)	316 (31.66%)	49 (31.21%)	
Symptom, %				0.549
No	819 (70.91%)	704 (70.54%)	115 (73.25%)	
Yes	336 (29.09%)	294 (29.46%)	42 (26.75%)	
Height, cm	166.21 (11.55)	166.26 (11.51)	165.89 (11.81)	0.713
Weight, kg	67.35 (13.50)	67.28 (13.21)	67.81 (15.25)	0.684
BMI, kg/m^2^	24.63 (7.31)	24.57 (7.73)	25.05 (3.70)	0.211
SBP, mmHg	143.06 (23.88)	142.55 (23.57)	146.34 (25.57)	0.083
DBP, mmHg	83.27 (14.87)	83.07 (14.39)	84.55 (17.60)	0.317
Smoke, %				0.371
No	635 (54.98%)	543 (54.41%)	92 (58.60%)	
Yes	520 (45.02%)	455 (45.59%)	65 (41.40%)	
Drunk, %				**0.013**
No	710 (61.47%)	628 (62.93%)	82 (52.23%)	
Yes	445 (38.53%)	370 (37.07%)	75 (47.77%)	
Diabetes, %				0.71
No	828 (71.69%)	713 (71.44%)	115 (73.25%)	
Yes	327 (28.31%)	285 (28.56%)	42 (26.75%)	
Hypertension, %				0.906
No	450 (38.96%)	390 (39.08%)	60 (38.22%)	
Yes	705 (61.04%)	608 (60.92%)	97 (61.78%)	
Operation History, %				** < 0.001**
No	727 (62.94%)	653 (65.43%)	74 (47.13%)	
Yes	428 (37.06%)	345 (34.57%)	83 (52.87%)	
History of Stroke, %				**0.006**
No	796 (68.92%)	703 (70.44%)	93 (59.24%)	
Yes	359 (31.08%)	295 (29.56%)	64 (40.76%)	
Operation Mode, %				0.053
No surgery	1086 (94.03%)	943 (94.49%)	143 (91.08%)	
CEA	16 (1.39%)	15 (1.50%)	1 (0.64%)	
CAS	53 (4.59%)	40 (4.01%)	13 (8.28%)	
Carotid artery ultrasound				
Maximum plaque diameter, cm	1.33 (0.90)	1.32 (0.89)	1.35 (0.95)	0.768
The branch with the largest plaque:				0.929
CCA	897 (77.66%)	776 (77.76%)	121 (77.07%)	
ICA	258 (22.34%)	222 (22.24%)	36 (22.93%)	
The direction with the largest plaque:				**0.035**
Left	510 (44.16%)	428 (42.89%)	82 (52.23%)	
Right	645 (55.84%)	570 (57.11%)	75 (47.77%)	
Maximum plaque length, cm	1.32 (0.88)	1.31 (0.88)	1.34 (0.91)	0.749
Maximum plaque width, cm	0.27 (0.11)	0.26 (0.10)	0.29 (0.13)	**0.026**
Maximum plaque area, cm^2^	0.38 (0.38)	0.38 (0.37)	0.43 (0.43)	0.19
LCCA plaque, %				1
No	312 (27.01%)	270 (27.05%)	42 (26.75%)	
Yes	843 (72.99%)	728 (72.95%)	115 (73.25%)	
LICA plaque, %				0.849
No	813 (70.39%)	704 (70.54%)	109 (69.43%)	
Yes	342 (29.61%)	294 (29.46%)	48 (30.57%)	
LECA plaque, %				0.244
No	1024 (88.66%)	880 (88.18%)	144 (91.72%)	
Yes	131 (11.34%)	118 (11.82%)	13 (8.28%)	
RCCA plaque, %				0.413
No	390 (33.77%)	342 (34.27%)	48 (30.57%)	
Yes	765 (66.23%)	656 (65.73%)	109 (69.43%)	
RICA plaque, %				0.943
No	847 (73.33%)	731 (73.25%)	116 (73.89%)	
Yes	308 (26.67%)	267 (26.75%)	41 (26.11%)	
RECA plaque, %				0.677
No	1030 (89.18%)	892 (89.38%)	138 (87.90%)	
Yes	125 (10.82%)	106 (10.62%)	19 (12.10%)	
Laboratory values				
INR	1.09 (0.65)	1.10 (0.69)	1.09 (0.23)	0.891
FIB, g/L	3.21 (7.63)	3.24 (8.21)	3.00 (0.85)	0.353
APTT, S	30.84 (7.96)	30.86 (8.28)	30.66 (5.59)	0.691
D dimer, ng/ml	251.97 (659.17)	240.96 (613.68)	321.99 (895.35)	0.275
ALT, U/L	26.41 (133.95)	26.95 (143.96)	22.99 (16.05)	0.402
AST, U/L	22.61 (11.40)	22.65 (11.12)	22.37 (13.09)	0.796
TBIL, umol/L	14.27 (6.98)	14.33 (6.58)	13.90 (9.10)	0.569
DBIL, umol/L	2.78 (2.31)	2.75 (1.76)	2.97 (4.45)	0.531
IBIL, umol/L	11.51 (5.54)	11.59 (5.52)	10.97 (5.66)	0.205
TP, g/L	65.85 (7.15)	66.04 (6.95)	64.65 (8.23)	**0.047**
ALB, g/L	38.65 (4.33)	38.75 (4.30)	38.02 (4.50)	0.061
GLB, g/L	27.74 (12.86)	27.83 (13.71)	27.18 (4.71)	0.255
UREA, mmol/L	6.52 (25.52)	6.61 (27.44)	5.95 (2.29)	0.453
SCR, umol/L	72.42 (47.55)	72.31 (49.23)	73.10 (35.15)	0.807
UA, umol/L	327.20 (96.60)	328.14 (97.72)	321.22 (89.16)	0.373
K, mmol/L	3.97 (0.39)	3.96 (0.39)	3.98 (0.43)	0.648
Na, mmol/L	139.59 (5.13)	139.61 (5.40)	139.45 (2.89)	0.566
Cl, mmol/L	105.75 (4.94)	105.78 (5.10)	105.59 (3.71)	0.59
Ca, mmol/L	2.55 (4.81)	2.59 (5.18)	2.26 (0.15)	**0.043**
HCY, umol/L	16.80 (13.87)	17.07 (14.44)	15.04 (9.40)	**0.021**
TC, mmol/L	5.00 (15.72)	4.83 (13.82)	6.09 (24.60)	0.532
TG, mmol/L	1.86 (10.44)	1.91 (11.22)	1.56 (1.05)	0.33
HDL-C, mmol/L	1.24 (2.72)	1.26 (2.92)	1.17 (0.27)	0.337
LDL-C, mmol/L	2.50 (3.86)	2.56 (4.13)	2.12 (0.80)	**0.003**
GLU, mmol/L	6.50 (2.82)	6.53 (2.76)	6.33 (3.19)	0.476
ESR, mm/H	15.29 (18.06)	14.81 (17.33)	18.33 (21.96)	0.057
WBC, *10^9^/L	7.58 (15.80)	7.59 (16.95)	7.51 (3.11)	0.898
RBC, *10^12^/L	5.26 (17.70)	5.39 (19.03)	4.44 (0.61)	0.116
Hb, g/L	140.82 (19.54)	141.15 (19.35)	138.73 (20.67)	0.171
PLT, *10^9^/L	219.53 (84.56)	220.64 (84.05)	212.50 (87.66)	0.278
LYM, *10^9^/L	2.07 (6.12)	2.13 (6.58)	1.73 (0.66)	0.062
MONO, *10^9^/L	0.48 (0.18)	0.48 (0.18)	0.48 (0.18)	0.571
NEUT, *10^9^/L	5.56 (23.12)	5.69 (24.86)	4.76 (2.40)	0.25
EO, *10^9^/L	0.15 (0.18)	0.14 (0.14)	0.20 (0.35)	**0.034**
BASO, *10^9^/L	0.06 (1.00)	0.07 (1.08)	0.03 (0.03)	0.298
LYMR, %	27.26 (9.74)	27.42 (9.70)	26.26 (9.95)	0.174
MONOR, %	7.28 (4.09)	7.27 (3.83)	7.40 (5.45)	0.769
NEUTR, %	62.78 (11.99)	62.69 (12.05)	63.36 (11.63)	0.502
EOSR, %	2.25 (2.67)	2.19 (2.62)	2.63 (2.98)	0.081
BASOR, %	0.45 (0.36)	0.45 (0.36)	0.43 (0.31)	0.491
Medications				
Antiplatelet, %				0.428
No	728 (63.03%)	634 (63.53%)	94 (59.87%)	
Yes	427 (36.97%)	364 (36.47%)	63 (40.13%)	
Aspirin, %				0.611
No	337 (29.18%)	288 (28.86%)	49 (31.21%)	
Yes	818 (70.82%)	710 (71.14%)	108 (68.79%)	
Clopidogrel, %				0.913
No	641 (55.50%)	555 (55.61%)	86 (54.78%)	
Yes	514 (44.50%)	443 (44.39%)	71 (45.22%)	
Hypoglycemic, %				**0.001**
No	972 (84.16%)	853 (85.47%)	119 (75.80%)	
Metformin + Acarbose	150 (12.99%)	115 (11.52%)	35 (22.29%)	
Insulin	33 (2.86%)	30 (3.01%)	3 (1.91%)	
Metformin, %				0.332
No	974 (84.33%)	837 (83.87%)	137 (87.26%)	
Yes	181 (15.67%)	161 (16.13%)	20 (12.74%)	
Acarbose, %				0.291
No	995 (86.15%)	855 (85.67%)	140 (89.17%)	
Yes	160 (13.85%)	143 (14.33%)	17 (10.83%)	
Antihypertensive, %				** < 0.001**
No	908 (78.61%)	815 (81.66%)	93 (59.24%)	
CCB + BBs	204 (17.66%)	140 (14.03%)	64 (40.76%)	
CCB + ACEI/ARBs	43 (3.72%)	43 (4.31%)	0 (0.00%)	
CCBs:				** < 0.001**
No	908 (78.61%)	815 (81.66%)	93 (59.24%)	
Yes	247 (21.39%)	183 (18.34%)	64 (40.76%)	
BBs:				** < 0.001**
No	951 (82.34%)	858 (85.97%)	93 (59.24%)	
Yes	204 (17.66%)	140 (14.03%)	64 (40.76%)	
ACEI/ARBs:				**0.015**
No	1112 (96.28%)	955 (95.69%)	157 (100.00%)	
Yes	43 (3.72%)	43 (4.31%)	0 (0.00%)	
Nifedipine, %				**0.01**
No	807 (69.87%)	683 (68.44%)	124 (78.98%)	
Yes	348 (30.13%)	315 (31.56%)	33 (21.02%)	
Hydrochlorothiazide, %				0.431
No	1073 (92.90%)	930 (93.19%)	143 (91.08%)	
Yes	82 (7.10%)	68 (6.81%)	14 (8.92%)	
Metoprolol, %				0.901
No	1008 (87.27%)	870 (87.17%)	138 (87.90%)	
Yes	147 (12.73%)	128 (12.83%)	19 (12.10%)	
Valsartan, %				0.672
No	1016 (87.97%)	880 (88.18%)	136 (86.62%)	
Yes	139 (12.03%)	118 (11.82%)	21 (13.38%)	
Antiplatelet Hypoglycemic, %				0.831
No	865 (74.89%)	749 (75.05%)	116 (73.89%)	
Yes	290 (25.11%)	249 (24.95%)	41 (26.11%)	
Hypoglycemic Antihypertensive, %				0.545
No	922 (79.83%)	800 (80.16%)	122 (77.71%)	
Yes	233 (20.17%)	198 (19.84%)	35 (22.29%)	
Antihypertensive Antiplatelet, %				0.386
No	673 (58.27%)	587 (58.82%)	86 (54.78%)	
Yes	482 (41.73%)	411 (41.18%)	71 (45.22%)	

Symptom indicates transient ischemic attack (Weakness, numbness or paralysis on one side of your body; Slurred speech or difficulty understanding others; Blindness in one or both eyes; Dizziness; Severe headache with no apparent cause).

*BMI* body mass index, *SBP* systolic blood pressure, *DBP* diastolic blood pressure.

Operation History, Any surgical procedure in the past 3 years (grade II and above).

*CEA* carotid endarterectomy, *CAS* carotid artery stenting, *LCCA* left common carotid artery, *LICA*, left internal carotid artery, *LECA* left external carotid artery, *RCCA* reft common carotid artery, *RICA* reft internal carotid artery, *RECA* reft external carotid artery, *INR* international normalized ratio, *FIB* fibrinogen, *APTT* activated partial thromboplastin time, *ALT* alamine aminotransferase, *AST* aspartate aminotransferase, *TBIL* serum total bilirubin, *DBIL* serum direct bilirubin, *IBIL* serum indirect bilirubin, *TP* serum total protein, *ALB* serum albumin, *GLB* serum globulin, *UREA* serum urea, *SCR* serum creatinine, *UA* uric acid, *K* kalium, *Ca* calcium, *Na* sodium, *Cl* chlorine, *HCY* homocysteine, *TC* total cholesterol, *TG* triglyceride, *HDL-C* high-density lipoprotein cholesterol, *LDL-C* low-density lipoprotein cholesterol, *GLU* fasting blood-glucose, *ESR* erythrocyte sedimentation rate, *WBC* white blood cell count, *RBC* red blood cell count, *Hb* haemoglobin, *PLT* blood platelet count, *LYM/LYMR* lymphocyte counts/proportion of lymphocytes, *MONO/MONOR* monocyte counts/proportion of monocyte, *NEUT/NEUTR* neutrophile counts/proportion of neutrophile granulocyte, *EO/EOSR* eosinophilic counts/proportion of eosinophilic granulocyte, *BASO/BASOR* basophilic counts/proportion of basophilic granulocyte, *CCBs* calcium channel blockers, *BBs* β-blockers, *ACEI/ARBs* angiotensin-converting enzyme inhibitors/angiotensin receptor blockers.

Significant values are in bold.

MACCE occurred in 157 patients within 3 years after the diagnosis of carotid stenosis (total events 13.59%, TIA or stroke 8.57%, AMI 3.29%, and mortality 3.46%). Compared with the control group, the MACCE group had a significantly higher proportion of patients with a history of drinking alcohol, previous 3-year operation history, and previous history of stroke. In addition, this group had higher mean maximum plaque width and eosinophil counts(EO), and a higher proportion of patients were on hypoglycemic and antihypertensive medications. At the same time, their total protein levels(TP), blood calcium concentrations(Ca), homocysteine levels(HCY), and low-density lipoprotein cholesterol (LDL.C) levels were relatively low. Notably, the largest plaques in patients who developed MACCE were more commonly found on the left side of the body. Baseline data showed no significant difference between the two groups in the prevalence rates of hyperglycemia and hypertension. However, patients who experienced MACCE required medication more frequently, reflecting the possibility that they may have poorer control of their diseases and thus be an important risk factor for the development of MACCE. In contrast to previous studies^28^, however, LDL.C levels were lower in the MACCE group, a difference that may be related to the smaller sample size in our study (Table 1).

**Fig. 2:**
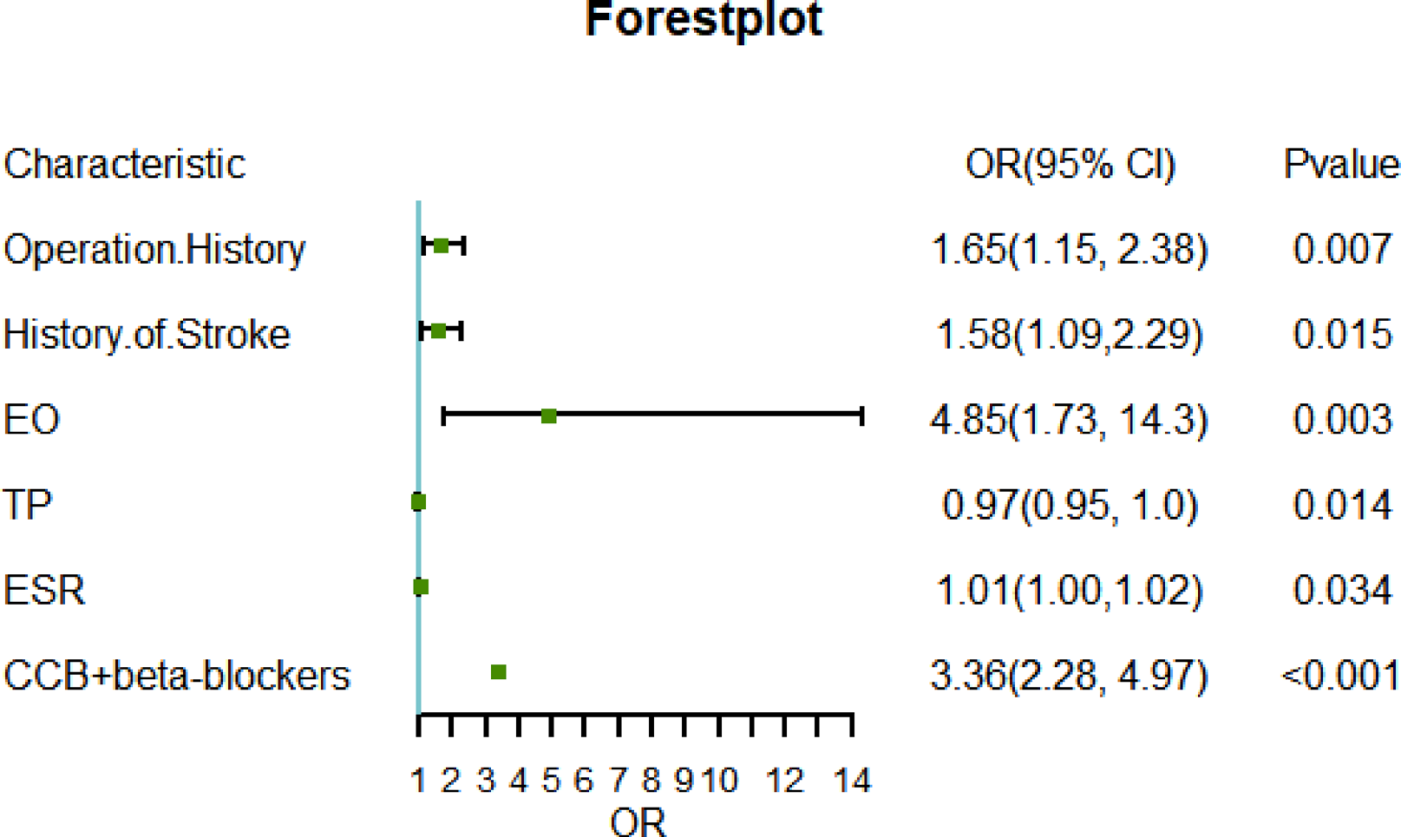
Factors associated with 3-year MACCE. Adjusted odds ratios for 3-year MACCE in the presence of associated factors during baseline Age, Gender, BMI, SBP, Smoke, Drunk, Diabetes, Hypertension.

The univariate logistic regression results showed that a history of drinking alcohol (OR 1.55 [1.11–2.18], *p* = 0.011), previous 3-year operation history (OR 2.12 [1.51–2.98], *p* < 0.001), previous history of stroke (OR 1.64[1.16–2.32], *p* = 0.005), history of carotid artery stenting (OR 2.14 [1.12–4.11], *p* = 0.021), the largest plaque was located on the right side (OR 0.69 [0.49–0.96], *p* = 0.029), maximum plaque width (OR 6.04 [1.54–23.71], *p* = 0.01), total proteins (TP) (OR 0.98 [0.95–1.00], *p* = 0.025), LDL.C(OR 0.76 [0.61–0.96], *p* = 0.019), erythrocyte sedimentation rate(ESR)(OR 1.01 [1.00–1.02], *p* = 0.025), EO(OR 4.03 [1.65–9.84], *p* = 0.002), treated with metformin and Acarbose (OR 2.18 [1.43–3.33], *p* < 0.001), treated with CCBs and BBs(OR 4.01 [2.78–5.77], *p* < 0.001), and treated with nifedipine (OR 0.58 [0.38–0.87], *p* = 0.008) were significantly associated with the occurrence of MACCE (Table 2). After multifactorial adjustment, previous 3-year operation history (OR 1.66[1.14 ~ 2.41], *p* = 0.008), previous history of stroke (OR 1.59 [1.10 ~ 2.31], *p* = 0.014), TP(OR 0.97 (0.95 ~ 0.99), *p* = 0.02), ESR(OR 1.01[1.00–1.02], *p* = 0.031), EO(OR 4.62 (1.60 ~ 13.35), *p* = 0.005) and treated with CCBs and BBs (OR 3.08 [2.04 ~ 4.63], *P* < 0.001) were identified as independent risk factors for long-term prognosis in patients with carotid artery stenosis(Fig. 2).

**Table 2:** Independent predictors of MACCE.

Factor	Level	Univariable	Multivariate
		OR (95% CI, P value)	OR (95% CI, P value)
Age		1.00 (0.98–1.02, *p* = 0.928)	
Gender	Man		
	Woman	0.98 (0.68–1.41, *p* = 0.910)	
Symptom	No		
	Yes	0.87 (0.60–1.28, *p* = 0.488)	
SBP		1.01 (1.00–1.01, *p* = 0.065)	
DBP		1.01 (1.00–1.02, *p* = 0.246)	
Height		1.00 (0.98–1.01, *p* = 0.707)	
Weight		1.00 (0.99–1.02, *p* = 0.650)	
BMI		1.01 (0.99–1.03, *p* = 0.454)	
Smoke	No		
	Yes	0.84 (0.60–1.19, *p* = 0.327)	
Drunk	No		
	Yes	**1.55 (1.11–2.18, *p* = 0.011)**	
Diabetes	No		
	Yes	0.91 (0.63–1.33, *p* = 0.641)	
Hypertension	No		
	Yes	1.04 (0.73–1.47, *p* = 0.837)	
Operation History	No		
	Yes	**2.12 (1.51–2.98, *p* < .001)**	**1.66 (1.14 ~ 2.41, *p* < .008)**
History of Stroke	No		
	Yes	**1.64 (1.16–2.32, *p* = 0.005)**	**1.59 (1.10 ~ 2.31, *p* < .014)**
Operation Mode	No		
	CEA	0.44 (0.06–3.35, *p* = 0.428)	
	CAS	2.14 (1.12–4.11, *p* = 0.051)	
Carotid artery ultrasound			
Maximum plaque diameter		1.03 (0.86–1.24, *p* = 0.757)	
The branch with the largest plaque	Left		
	Right	1.04 (0.70–1.55, *p* = 0.848)	
The direction with the largest plaque	Left		
	Right	**0.69 (0.49–0.96, *p* = 0.029)**	
Maximum plaque length		1.03 (0.86–1.25, *p* = 0.743)	
Maximum plaque width		**6.04 (1.54–23.71, *p* = 0.010)**	
Maximum plaque area		1.35 (0.90–2.02, *p* = 0.147)	
LCCA	No		
	Yes	1.02 (0.69–1.48, *p* = 0.937)	
LICA	No		
	Yes	1.05 (0.73–1.52, *p* = 0.776)	
LECA	No		
	Yes	0.67 (0.37–1.23, *p* = 0.196)	
RCCA	No		
	Yes	1.18 (0.82–1.70, *p* = 0.363)	
RICA	No		
	Yes	0.97 (0.66–1.42, *p* = 0.866)	
RECA	No		
	Yes	1.16 (0.69–1.95, *p* = 0.579)	
Laboratory values			
INR		0.99 (0.75–1.30, *p* = 0.944)	
FIB		0.99 (0.92–1.07, *p* = 0.774)	
APTT		1.00 (0.97–1.02, *p* = 0.763)	
D dimer		1.00 (1.00–1.00, *p* = 0.170)	
ALT		1.00 (1.00–1.00, *p* = 0.764)	
AST		1.00 (0.98–1.01, *p* = 0.771)	
TBIL		0.99 (0.96–1.02, *p* = 0.471)	
DBIL		1.03 (0.98–1.09, *p* = 0.284)	
IBIL		0.98 (0.95–1.01, *p* = 0.195)	
TP		**0.98 (0.95–1.00, *p* = 0.025)**	**0.97 (0.95 ~ 0.99, *p* = 0.020)**
ALB		0.96 (0.93–1.00, *p* = 0.051)	
GLB		0.99 (0.98–1.01, *p* = 0.558)	
UREA		1.00 (0.99–1.01, *p* = 0.781)	
SCR		1.00 (1.00–1.00, *p* = 0.848)	
UA		1.00 (1.00–1.00, *p* = 0.404)	
K		1.11 (0.72–1.71, *p* = 0.624)	
Na		0.99 (0.97–1.02, *p* = 0.710)	
Cl		0.99 (0.96–1.02, *p* = 0.668)	
Ca		0.50 (0.13–1.91, *p* = 0.313)	
HCY		0.99 (0.97–1.00, *p* = 0.091)	
TC		1.00 (1.00–1.01, *p* = 0.374)	
TG		0.99 (0.90–1.08, *p* = 0.809)	
HDL.C		0.96 (0.75–1.24, *p* = 0.778)	
LDL.C		0.76 (0.61–0.96, *p* = 0.019)	
GLU		0.97 (0.91–1.04, *p* = 0.429)	
ESR		**1.01 (1.00–1.02, *p* = 0.025)**	**1.01(1.00–1.02, *p* = 0.031)**
WBC		1.00 (0.99–1.01, *p* = 0.955)	
RBC		0.80 (0.61–1.05, *p* = 0.112)	
Hb		0.99 (0.99–1.00, *p* = 0.149)	
PLT		1.00 (1.00–1.00, *p* = 0.259)	
LYM		0.81 (0.64–1.04, *p* = 0.095)	
MONO		1.30 (0.52–3.23, *p* = 0.574)	
NEUT		1.00 (0.97–1.02, *p* = 0.686)	
EO		**4.03 (1.65–9.84, *p* = 0.002)**	**4.62 (1.60 ~ 13.35, *p* = 0.005)**
BASO		0.74 (0.06–8.71, *p* = 0.814)	
LYMR		0.99 (0.97–1.01, *p* = 0.165)	
MONOR		1.01 (0.97–1.05, *p* = 0.707)	
NEUTR		1.00 (0.99–1.02, *p* = 0.512)	
EOSR		1.05 (1.00–1.10, *p* = 0.072)	
BASOR		0.86 (0.52–1.40, *p* = 0.537)	
Medications			
Antiplatelet Hypoglycemic	No		
	Yes	1.06 (0.72–1.56, *p* = 0.754)	
Aspirin	No		
	Yes	0.89 (0.62–1.29, *p* = 0.547)	
Clopidogrel	No		
	Yes	1.03 (0.74–1.45, *p* = 0.845)	
Antiplatelet	No		
	Yes	1.17 (0.83–1.65, *p* = 0.378)	
Hypoglycemic	No		
	Metformin + Acarbose	**2.18 (1.43–3.33, *p* < 0.001)**	
	Insulin	0.72 (0.22–2.38, *p* = 0.587)	
Hypoglycemic Antihypertensive	No		
	Yes	1.16 (0.77–1.74, *p* = 0.477)	
Metformin	No		
	Yes	0.76 (0.46–1.25, *p* = 0.278)	
Acarbose	No		
	Yes	0.73 (0.43–1.24, *p* = 0.240)	
Antihypertensive	No		
	CCB + BBs	**4.01 (2.78–5.77, *p* < 0.001)**	**3.08 (2.04 ~ 4.63, *p* < 0.001)**
	CCB + ACEI/ARBs	0.00 (0.00-Inf, *p* = 0.980)	
CCBs	No		
	Yes	**3.06 (2.15–4.38, *p* < 0.001)**	
BBs	No		
	Yes	**4.22 (2.93–6.08, *p* < 0.001)**	
ACEI/ARBs	No		
	Yes	0.00 (0.00-Inf,* p* = 0.979)	
Antihypertensive Antiplatelet	No		
	Yes	1.18 (0.84–1.65, *p* = 0.340)	
Nifedipine	No		
	Yes	**0.58 (0.38–0.87, *p* = 0.008)**	
Hydrochlorothiazide	No		
	Yes	1.34 (0.73–2.44, *p* = 0.342)	
Metoprolol	No		
	Yes	0.94 (0.56–1.57, *p* = 0.800)	
Valsartan	No		
	Yes	1.15 (0.70–1.89, *p* = 0.579)	

Each factor was analyzed separately, and then factors independently associated with MACCE were selected and included in the multivariate analysis (*P* < 0.05).

*CI* confidence interval.

Significant values are in bold.

**Fig. 3:**
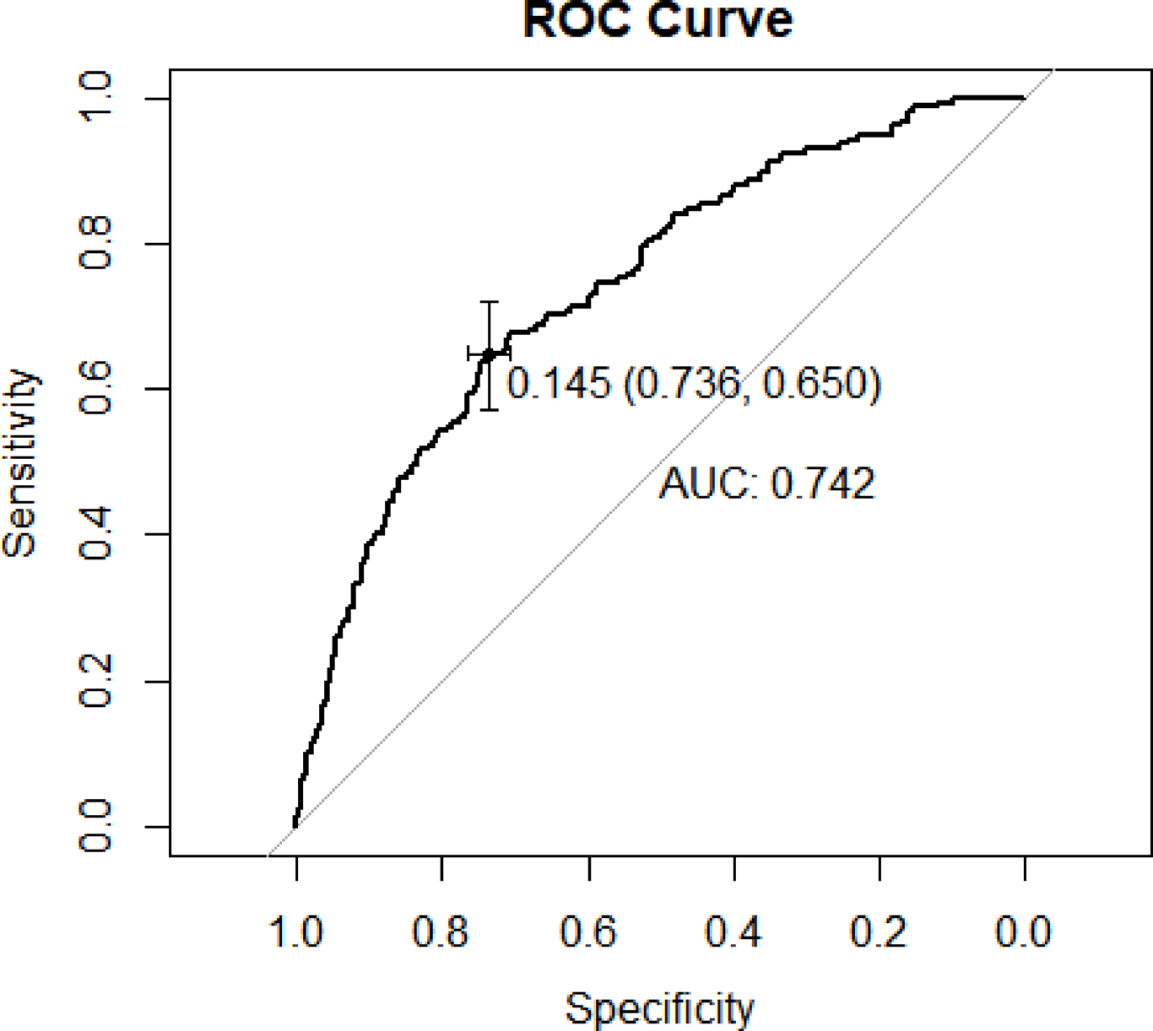
AUROC of the regression model based on Operation history, previous history of stroke, TP, ESR, EO and treated with CCBs and BBs in the full dataset.

**Fig. 4:**
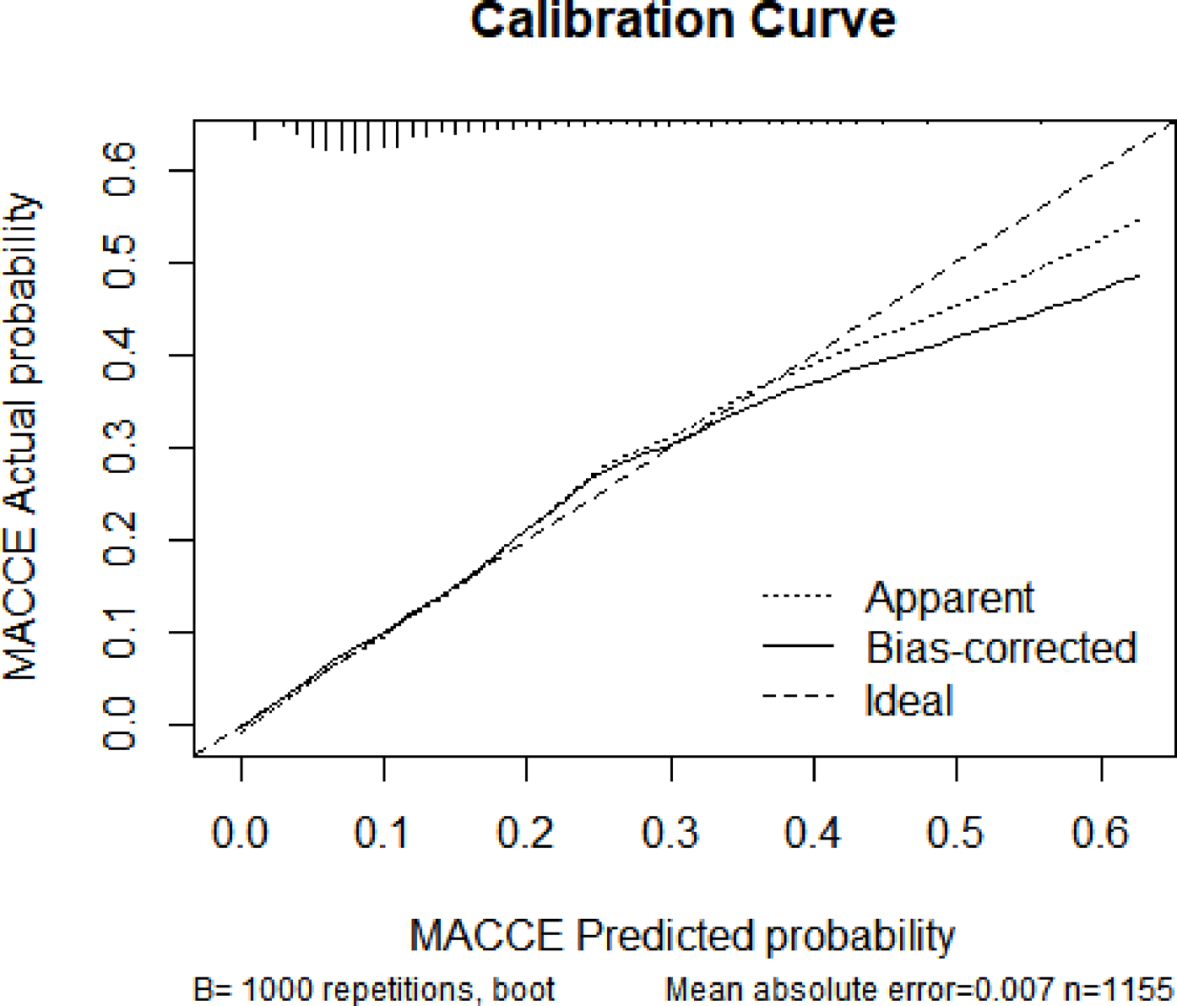
Calibration curve of regression model based on Operation history, previous history of stroke, TP, ESR, EO and treated with CCBs and BBs in the full dataset.

The logistic regression model, which incorporated previous operation history, previous history of stroke, TP, ESR, EO and treated with CCBs and BBs and traditional risk factors exhibited good performance in identifying the occurrence of MACCE in patients diagnosed with carotid stenosis, with an ROC of 0.739 [95% CI 0.6977–0.7805], a sensitivity of 64.3%, and a specificity of 72.4% (Fig. 3). The calibration curve results showed a high degree of overlap between the corrected curve and the ideal curve, which implied that the model prediction results were consistent with the actual occurrence of the events in patients (Fig. 4). For the internal validation of the model, the strengthen Boostrap method was used to resample the full dataset cohort 1000 times to calculate the high estimate of the logistic regression model. The results showed a high estimate of 0.0185 for the ROC and − 0.0013 for the Brier score. After imputing the final model performance correction, the real model performed well with an ROC of 0.721 and a Brier score of 0.0539 (Table 3).

**Table 3:** ROC and Brier scores for internal validation of models by strengthen bootstrap 1000 resampling times.

	Original dataset	Training dataset	Test dataset	Optimism	Original dataset corrected
ROC	0.739	0.749	0.73	0.0185	0.721
Brier	0.05255	0.05195	0.0533	− 0.0013	0.0539

Using the receiver operating characteristic (ROC) curve, we determined the optimal cutoff value for each significant univariate continuous variable with the Youden index (Supplementary Table 2). In our model analysis, the EO had the highest OR value of 4.62. To explore in depth the potential of the EO in predicting MACCE, we categorized the EO into two groups on the basis of its optimal cutoff value, and thus assessed its efficacy in predicting the primary and secondary outcomes. After adjustment for traditional risk factors such as age, sex, BMI, SBP, smoking, drinking, diabetes, and hypertension, our results showed that EO was a significant risk factor in predicting both primary and secondary outcomes of our concerns. Although no statistically significant differences were observed between the two groups for acute myocardial infarction(AMI), this may be related to the low incidence of these events (Table 4). Next, we performed a dichotomous analysis of other potential risk factors to see their effect in predicting primary and secondary outcomes. The results of the study showed that patients treated with CCBs and BBs exhibited a significant increased risk for all outcomes. (Supplementary Table 3-7).

**Table 4:** Predictive value of the eosinophils counts for primary endpoint in univariate and multivariate analysis.

Variables	Events/subjects	Univariate analysis		Multivariate analysis	
		OR (95% CI)	*P* value	Adjusted OR (95% CI)	*P* value
MACCE					
EO < 0.185	100/755	Reference	–	Reference	–
EO > 0.185	57/243	1.77 (1.24–2.53)	*p* = 0.002	1.72 (1.20–2.46)	*p* = 0.003
TIA OR Stroke					
EO < 0.185	59/796	Reference	–	Reference	–
EO > 0.185	40/260	2.08 (1.36–3.18)	*p* < 0.001	2.02 (1.31–3.11)	*p* = 0.002
AMI					
EO < 0.185	24/831	Reference	–	Reference	–
EO > 0.185	14/286	1.69 (0.86–3.32)	*p* = 0.124	1.78 (0.90–3.53)	*p* = 0.096

## Discussion

The severity of ASCVD in public health has been extensively studied and recognized^29^. The inflammatory response of the arterial wall tissue characterized by chronic progressive inflammation and the formation of sclerotic plaques plays a crucial role in pathogenesis^30^. The complications associated with atherosclerosis are even more critical in the carotid artery, which is the primary blood supply vessel to the brain, and its impact on cerebral perfusion provides the opportunity for stroke, myocardial infarction, and peripheral arterial disease. To explore the key factors affecting the prognosis of patients with carotid stenosis, multidimensional data, including demographic characteristics, serum laboratory measurements, and imaging features, were fully utilized. Previous studies have shown that multiple factors differ regarding their effect on the prognosis of patients with carotid stenosis. However, eosinophils, as a terminally differentiated leukocyte in the body, has not been studied thoroughly in the prognosis of carotid stenosis.

Atherosclerosis is a chronic inflammatory response whose progression is closely linked to endothelial dysfunction as well as oxidative stress^31^. As part of the innate immune system, eosinophils have been shown to be involved in the development of atherosclerosis and have been recognized as a promising biomarker^32,33^. In addition, the high expression levels of eosinophil chemokines in atherosclerotic lesions further support this finding^34^. Despite the fact that eosinophils contain cytokines with anti-inflammatory effects such as IL4 and IL13, the experimental results of Meng et al.^20^ showed that the knockout of the IL4 and IL13 genes was not sufficient to accelerate atherosclerosis development. Instead, their study revealed the important role of ECP in promoting smooth muscle cell (SMC) calcification as well as atherosclerotic progression^20^.

The role of eosinophils in cardiovascular disease has garnered widespread attention^35^, Studies have demonstrated that elevated eosinophil levels can release various cationic proteins and generate extracellular traps, thereby promoting the initiation and propagation of inflammatory responses^35,36^. Surfactant protein-D (SP-D), secreted by airway epithelial cells, can inhibit the formation of eosinophil extracellular traps. However, SP-D is inherently susceptible to oxidation, and once oxidized, its ability to suppress extracellular trap formation is markedly diminished^37^. Moreover, genetic markers associated with eosinophilia may also contribute to the pathogenesis of cardiovascular diseases. Several microRNAs, such as miR-10a, miR-125, miR-21, and miR-233, not only play regulatory roles in eosinophil-related inflammatory diseases^38–40^, but also participate in multiple key processes of atherosclerosis, including the expression of endothelial adhesion molecules, foam cell formation, macrophage polarization, and ventricular remodeling following myocardial infarction^41,42^. These findings suggest that eosinophil-mediated immune regulatory mechanisms may be involved in the development and progression of carotid and coronary atherosclerosis through multiple pathways, warranting further in-depth investigation. Thrombosis, a serious complication of atherosclerosis, has attracted a great deal of attention from researchers, and a study by Stefan Uderhardt et al.^43^ points out that eosinophils play a key role in promoting thrombosis. Studies have shown that in atherosclerosis complicated by thrombosis, eosinophils are activated by being attracted to the edge of the thrombus and interacting with platelets. This activation process leads to the production of eosinophil extracellular traps (EETs), which exacerbates the inflammatory response and thus promotes the deterioration of atherosclerosis. Also, EETs further activate platelets and exacerbate thrombus formation. These findings point to the fact that eosinophils may be a potential target for future treatment of atherosclerosis and its thrombotic complications^16^.

There are conflicting views in the field of clinical research on eosinophils and MACCE. Meng et al.^20^ reported a strong connection between eosinophil counts and levels of eosinophil cationic protein and coronary artery calcification in a randomized, controlled clinical screening experiment that included 5864 individuals with coronary artery disease. Furthermore, the results of two earlier extensive clinical studies were in agreement^44,45^. However, some researchers have reached contradictory conclusions, and their findings suggest that eosinophils actually play a protective role in cardiovascular disease. Gao et al.^46^ recruited 5,287 patients who received coronary angiograms and measured their biochemical markers in a study. The results of the study showed that the percentage of eosinophils in leukocytes showed a significant negative correlation with the degree of coronary artery stenosis and the occurrence of acute myocardial infarction. Similarly, a study by Guner et al.^22^ found that those patients with a low eosinophil ratio on admission were more likely to experience MACE during hospitalization. In the current controversy over the role of eosinophils in CVD, Norbert Gerdes makes the point that in stable CVD, higher levels of eosinophils may predict the risk of future adverse events; and in the case of acute injury, diseased tissue may reduce the number of blood cells detectable by recruiting eosinophils. In our study, after adjusting for traditional risk factors, those patients who experienced MACCE showed a significant increase in the number of eosinophils in the peripheral blood compared to those who did not. (OR 1.72 [1.20–2.46], *p* = 0.003). This is consistent with Norbert Gerdes’s point.

In addition to eosinophils, previous 3-year operation history, history of stroke, TP, ESR, treated with CCBs and BBs was also significantly associated with MACCE events at 3 years. By applying multifactor logistic regression analysis, we constructed a model for the prediction of MACCE over a 3-year period. The model had a ROC curve area value of 0.739, 0.721 after adjustment, indicating a high predictive accuracy. Notably, although not shown to be statistically significant in AMI, EO was recognised as a risk factor after dichotomisation according to the cut-off values, both in the primary and secondary outcomes.

This study has several limitations. First, it is a single-center retrospective study with a relatively small sample size, which may introduce selection bias and limit the generalizability of the findings to other populations or countries. Second, due to the retrospective nature of the study, we were only able to identify an association between eosinophil levels and the occurrence of MACCE within 3 years, without establishing a causal relationship. Prospective, multi-center studies with larger cohorts are needed to further validate the reliability and robustness of our findings. Third, the follow-up period was limited to three years, and data regarding long-term outcomes were not available, which restricts our ability to predict long-term prognosis. Fourth, the study did not include the assessment of other potential risk factors such as genetic markers, lipidomic profiles and Socio-economic levels, which may have influenced the results. Despite these limitations, our findings demonstrate that eosinophil count is an independent risk factor for MACCE within three years in patients with carotid artery stenosis, providing important support for further investigations into the role of eosinophils in the pathogenesis of atherosclerosis.

## Conclusion

Eosinophil counts (> 0.185*10^9^/L) were independently associated with the occurrence of MACCE within 3 years in patients hospitalized for carotid stenosis, but there was no significant difference in proportion of eosinophils between the two groups. In addition, previous operation history, previous history of stroke, lower TP (< 59.95 g/L), higher ESR (> 15.5 mm/h) and treated with CCBs and BBs were also strongly associated with the occurrence of MACCE.

## Electronic supplementary material

Below is the link to the electronic supplementary material.

Supplementary Material 1

## Data Availability

The data and analytical methods are available from the corresponding authors on reasonable request. Supplementary Material has been uploaded separately on submission and would be available online.

## Abbreviations

CCBs: Calcium channel blockers
BBs: β-Blockers
MACCE: Major adverse cardiac and cerebrovascular event
MACE: Major adverse cardiovascular event
ASCVD: Atherosclerotic cardiovascular disease
TIA: Transient ischemic attack
AMI: Acute myocardial infarction
ROC: Receiver operating characteristic
EO: Eosinophil counts
ECP: Eosinophil cationic proteins
STEMI: ST-segment elevation myocardial infarction
LDL.C: Low-density lipoprotein cholesterol
TP: Total proteins
Ca: Calcium
HCY: Homocysteine
ESR: Erythrocyte sedimentation rate
SMC: Smooth muscle cell
EETs: Eosinophil extracellular traps
NASCET: North American symptomatic carotid endarterectomy trial
BMI: Body mass index
CEA: Carotid endarterectomy
CAS: Carotid artery stenting
ACEI: Angiotensin converting enzyme inhibitor
ARB: Angiotensin receptor blocker
CCA: Common carotid artery
ICA: Internal carotid artery
ECA: External carotid artery
