# Supplementary material for: Eosinophil counts predict 3-year risk of major adverse cardiovascular and cerebrovascular events in carotid artery stenosis patients
Source: Sci Rep. 2025 Jul 1;15:20939. doi: 10.1038/s41598-025-06350-w (PMC12217005; doi:10.1038/s41598-025-06350-w)
Supplement: Supplementary file 1 — Supplementary Material 1 [file 41598_2025_6350_MOESM1_ESM.docx]

Supplementary

|  | ALL | No MACCE | MACCE | p.value |
| --- | --- | --- | --- | --- |
|  | N=1155 | N=998 | N=157 |  |
| Age(mean±SD) | 65.09 (10.41) | 65.10 (10.42) | 65.01 (10.35) | 0.924 |
| Gender: |  |  |  | 1 |
| Man | 789 (68.37%) | 682 (68.34%) | 107 (68.59%) |  |
| Woman | 365 (31.63%) | 316 (31.66%) | 49 (31.41%) |  |
| Symptom: |  |  |  | 0.473 |
| No | 819 (70.97%) | 704 (70.54%) | 115 (73.72%) |  |
| Yes | 335 (29.03%) | 294 (29.46%) | 41 (26.28%) |  |
| Height | 165.60 (10.26) | 165.65 (10.38) | 164.95 (8.61) | 0.558 |
| Weight | 66.95 (12.30) | 66.98 (12.27) | 66.51 (12.79) | 0.791 |
| BMI | 24.60 (7.86) | 24.62 (8.08) | 24.19 (3.79) | 0.467 |
| SBP | 143.10 (23.91) | 142.55 (23.59) | 146.66 (25.67) | 0.063 |
| DBP | 83.32 (14.85) | 83.09 (14.38) | 84.81 (17.60) | 0.25 |
| Smoke: |  |  |  | 0.388 |
| No | 634 (55.18%) | 543 (54.63%) | 91 (58.71%) |  |
| Yes | 515 (44.82%) | 451 (45.37%) | 64 (41.29%) |  |
| Drunk: |  |  |  | 0.013 |
| No | 709 (61.65%) | 628 (63.12%) | 81 (52.26%) |  |
| Yes | 441 (38.35%) | 367 (36.88%) | 74 (47.74%) |  |
| Diabetes: |  |  |  | 0.631 |
| No | 822 (71.79%) | 707 (71.49%) | 115 (73.72%) |  |
| Yes | 323 (28.21%) | 282 (28.51%) | 41 (26.28%) |  |
| Hypertension: |  |  |  | 0.82 |
| No | 445 (38.97%) | 386 (39.15%) | 59 (37.82%) |  |
| Yes | 697 (61.03%) | 600 (60.85%) | 97 (62.18%) |  |
| Operation.History: |  |  |  | <0.001 |
| No | 723 (62.87%) | 649 (65.36%) | 74 (47.13%) |  |
| Yes | 427 (37.13%) | 344 (34.64%) | 83 (52.87%) |  |
| History.of.Stroke: |  |  |  | 0.008 |
| No | 793 (69.08%) | 700 (70.56%) | 93 (59.62%) |  |
| Yes | 355 (30.92%) | 292 (29.44%) | 63 (40.38%) |  |
| Operation.Mode: |  |  |  | 0.046 |
| No surgery | 1074 (94.05%) | 932 (94.52%) | 142 (91.03%) |  |
| CEA | 16 (1.40%) | 15 (1.52%) | 1 (0.64%) |  |
| CAS | 52 (4.55%) | 39 (3.96%) | 13 (8.33%) |  |
| Carotid artery ultrasound | | | | |
| Maximum.plaque.diameter(mean±SD) | 1.33 (0.85) | 1.32 (0.86) | 1.39 (0.78) | 0.424 |
| The.branch.with.the.largest.plaque: |  |  |  | 0.426 |
| CCA | 729 (81.09%) | 642 (80.65%) | 87 (84.47%) |  |
| ICA | 170 (18.91%) | 154 (19.35%) | 16 (15.53%) |  |
| The.direction.with.the.largest.plaque: |  |  |  | 0.157 |
| Left | 391 (43.49%) | 339 (42.59%) | 52 (50.49%) |  |
| Right | 508 (56.51%) | 457 (57.41%) | 51 (49.51%) |  |
| Maximum.plaque.length(mean±SD) | 1.31 (0.84) | 1.30 (0.85) | 1.38 (0.76) | 0.373 |
| Maximum.plaque.width(mean±SD) | 0.26 (0.10) | 0.26 (0.10) | 0.28 (0.14) | 0.132 |
| Maximum.plaque.area(mean±SD) | 0.37 (0.35) | 0.37 (0.35) | 0.41 (0.36) | 0.235 |
| LCCA: |  |  |  | 0.291 |
| No | 237 (25.90%) | 215 (26.51%) | 22 (21.15%) |  |
| Yes | 678 (74.10%) | 596 (73.49%) | 82 (78.85%) |  |
| LICA: |  |  |  | 0.515 |
| No | 688 (75.19%) | 613 (75.59%) | 75 (72.12%) |  |
| Yes | 227 (24.81%) | 198 (24.41%) | 29 (27.88%) |  |
| LECA: |  |  |  | 1 |
| No | 857 (93.66%) | 760 (93.71%) | 97 (93.27%) |  |
| Yes | 58 (6.34%) | 51 (6.29%) | 7 (6.73%) |  |
| RCCA: |  |  |  | 0.017 |
| No | 311 (33.99%) | 287 (35.39%) | 24 (23.08%) |  |
| Yes | 604 (66.01%) | 524 (64.61%) | 80 (76.92%) |  |
| RICA: |  |  |  | 0.868 |
| No | 729 (79.67%) | 645 (79.53%) | 84 (80.77%) |  |
| Yes | 186 (20.33%) | 166 (20.47%) | 20 (19.23%) |  |
| RECA: |  |  |  | 0.384 |
| No | 858 (93.77%) | 763 (94.08%) | 95 (91.35%) |  |
| Yes | 57 (6.23%) | 48 (5.92%) | 9 (8.65%) |  |
| Laboratory values | | | | |
| INR(mean±SD) | 1.10 (0.68) | 1.10 (0.72) | 1.09 (0.23) | 0.742 |
| FIB(mean±SD) | 3.22 (8.00) | 3.26 (8.61) | 2.98 (0.84) | 0.336 |
| APTT(mean±SD) | 30.88 (8.31) | 30.90 (8.66) | 30.73 (5.73) | 0.764 |
| D.dimer(mean±SD) | 244.44 (629.92) | 230.51 (565.81) | 329.40 (928.08) | 0.218 |
| ALT(mean±SD) | 26.62 (139.82) | 27.24 (150.61) | 22.78 (16.22) | 0.389 |
| AST(mean±SD) | 22.62 (11.63) | 22.62 (11.32) | 22.63 (13.45) | 0.993 |
| TBIL(mean±SD) | 14.54 (7.14) | 14.58 (6.71) | 14.32 (9.42) | 0.751 |
| DBIL(mean±SD) | 2.77 (2.42) | 2.73 (1.82) | 3.01 (4.69) | 0.484 |
| IBIL(mean±SD) | 11.57 (5.68) | 11.67 (5.67) | 10.95 (5.72) | 0.165 |
| TP(mean±SD) | 66.02 (7.18) | 66.24 (6.94) | 64.65 (8.41) | 0.035 |
| ALB(mean±SD) | 38.65 (4.25) | 38.75 (4.22) | 38.01 (4.38) | 0.061 |
| GLB(mean±SD) | 27.72 (12.24) | 27.83 (13.05) | 27.08 (4.77) | 0.211 |
| UREA(mean±SD) | 6.58 (26.76) | 6.68 (28.86) | 6.00 (2.33) | 0.491 |
| SCR(mean±SD) | 71.49 (43.42) | 71.21 (44.54) | 73.18 (35.98) | 0.552 |
| UA(mean±SD) | 327.02 (97.99) | 326.45 (98.55) | 331.16 (94.23) | 0.657 |
| K(mean±SD) | 3.96 (0.39) | 3.96 (0.38) | 3.98 (0.43) | 0.564 |
| Na(mean±SD) | 139.63 (5.30) | 139.66 (5.59) | 139.43 (2.95) | 0.451 |
| Cl(mean±SD) | 105.75 (5.07) | 105.78 (5.26) | 105.51 (3.71) | 0.438 |
| Ca(mean±SD) | 2.56 (5.07) | 2.61 (5.46) | 2.26 (0.15) | 0.054 |
| HCY(mean±SD) | 16.52 (13.01) | 16.72 (13.39) | 15.08 (9.83) | 0.118 |
| TC(mean±SD) | 4.52 (9.92) | 4.59 (10.63) | 4.08 (1.23) | 0.182 |
| TG(mean±SD) | 1.92 (11.33) | 1.97 (12.13) | 1.54 (1.09) | 0.316 |
| HDL.C(mean±SD) | 1.26 (2.95) | 1.27 (3.16) | 1.17 (0.26) | 0.363 |
| LDL.C(mean±SD) | 2.48 (3.33) | 2.53 (3.54) | 2.16 (0.83) | 0.011 |
| GLU(mean±SD) | 6.45 (2.71) | 6.45 (2.59) | 6.41 (3.41) | 0.884 |
| ESR(mean±SD) | 15.04 (17.32) | 14.75 (16.98) | 16.96 (19.48) | 0.287 |
| WBC(mean±SD) | 7.45 (16.26) | 7.49 (17.49) | 7.24 (2.66) | 0.689 |
| RBC(mean±SD) | 5.32 (18.29) | 5.46 (19.70) | 4.46 (0.61) | 0.121 |
| Hb(mean±SD) | 140.77 (19.55) | 141.14 (19.32) | 138.51 (20.85) | 0.15 |
| PLT(mean±SD) | 219.28 (84.15) | 219.96 (83.57) | 215.05 (87.80) | 0.523 |
| LYM(mean±SD) | 2.10 (6.32) | 2.15 (6.80) | 1.76 (0.64) | 0.083 |
| MONO(mean±SD) | 0.48 (0.18) | 0.47 (0.18) | 0.49 (0.18) | 0.414 |
| NEUT(mean±SD) | 5.64 (23.91) | 5.78 (25.73) | 4.77 (2.45) | 0.245 |
| EO(mean±SD) | 0.15 (0.18) | 0.14 (0.14) | 0.20 (0.36) | 0.039 |
| BASO(mean±SD) | 0.07 (1.03) | 0.07 (1.11) | 0.03 (0.03) | 0.29 |
| LYMR(mean±SD) | 27.36 (9.65) | 27.60 (9.65) | 25.88 (9.51) | 0.043 |
| MONOR(mean±SD) | 7.31 (4.19) | 7.30 (3.93) | 7.42 (5.58) | 0.802 |
| NEUTR(mean±SD) | 62.54 (11.96) | 62.38 (12.03) | 63.56 (11.46) | 0.253 |
| EOSR(mean±SD) | 2.29 (2.73) | 2.23 (2.68) | 2.66 (3.02) | 0.105 |
| BASOR(mean±SD) | 0.45 (0.35) | 0.45 (0.36) | 0.44 (0.31) | 0.568 |
| Medications | | | | |
| Antiplatelet: |  |  |  | 0.352 |
| No | 685 (64.68%) | 603 (65.26%) | 82 (60.74%) |  |
| Yes | 374 (35.32%) | 321 (34.74%) | 53 (39.26%) |  |
| Aspirin: |  |  |  | 0.891 |
| No | 300 (27.99%) | 262 (28.11%) | 38 (27.14%) |  |
| Yes | 772 (72.01%) | 670 (71.89%) | 102 (72.86%) |  |
| Clopidogrel: |  |  |  | 0.836 |
| No | 601 (56.75%) | 526 (56.93%) | 75 (55.56%) |  |
| Yes | 458 (43.25%) | 398 (43.07%) | 60 (44.44%) |  |
| Hypoglycemic: |  |  |  | 0.006 |
| No | 910 (84.26%) | 792 (85.53%) | 118 (76.62%) |  |
| Metformin+Acarbose | 142 (13.15%) | 109 (11.77%) | 33 (21.43%) |  |
| Insulin | 28 (2.59%) | 25 (2.70%) | 3 (1.95%) |  |
| Metformin: |  |  |  | 0.236 |
| No | 916 (84.81%) | 780 (84.23%) | 136 (88.31%) |  |
| Yes | 164 (15.19%) | 146 (15.77%) | 18 (11.69%) |  |
| Acarbose: |  |  |  | 0.318 |
| No | 937 (86.76%) | 799 (86.29%) | 138 (89.61%) |  |
| Yes | 143 (13.24%) | 127 (13.71%) | 16 (10.39%) |  |
| Antihypertensive: |  |  |  | <0.001 |
| No | 848 (78.45%) | 758 (81.59%) | 90 (59.21%) |  |
| CCBs+BBs | 197 (18.22%) | 135 (14.53%) | 62 (40.79%) |  |
| CCBs+ACEI/ARBs | 36 (3.33%) | 36 (3.88%) | 0 (0.00%) |  |
| CCBs: |  |  |  | <0.001 |
| No | 912 (79.65%) | 819 (82.73%) | 93 (60.00%) |  |
| Yes | 233 (20.35%) | 171 (17.27%) | 62 (40.00%) |  |
| BBs: |  |  |  | <0.001 |
| No | 952 (82.85%) | 859 (86.42%) | 93 (60.00%) |  |
| Yes | 197 (17.15%) | 135 (13.58%) | 62 (40.00%) |  |
| ACEI/ARBs: |  |  |  | 0.068 |
| No | 1006 (96.55%) | 912 (96.20%) | 94 (100.00%) |  |
| Yes | 36 (3.45%) | 36 (3.80%) | 0 (0.00%) |  |
| Nifedipine: |  |  |  | 0.011 |
| No | 766 (70.21%) | 644 (68.73%) | 122 (79.22%) |  |
| Yes | 325 (29.79%) | 293 (31.27%) | 32 (20.78%) |  |
| Hydrochlorothiazide: |  |  |  | 0.317 |
| No | 1016 (93.13%) | 876 (93.49%) | 140 (90.91%) |  |
| Yes | 75 (6.87%) | 61 (6.51%) | 14 (9.09%) |  |
| Metoprolol: |  |  |  | 1 |
| No | 954 (87.44%) | 819 (87.41%) | 135 (87.66%) |  |
| Yes | 137 (12.56%) | 118 (12.59%) | 19 (12.34%) |  |
| Valsartan: |  |  |  | 0.511 |
| No | 963 (88.27%) | 830 (88.58%) | 133 (86.36%) |  |
| Yes | 128 (11.73%) | 107 (11.42%) | 21 (13.64%) |  |
| Antiplatelet.Hypoglycemic: |  |  |  | 0.845 |
| No | 813 (75.63%) | 698 (75.79%) | 115 (74.68%) |  |
| Yes | 262 (24.37%) | 223 (24.21%) | 39 (25.32%) |  |
| Hypoglycemic.Antihypertensive: |  |  |  | 0.387 |
| No | 862 (80.79%) | 742 (81.27%) | 120 (77.92%) |  |
| Yes | 205 (19.21%) | 171 (18.73%) | 34 (22.08%) |  |
| Antihypertensive.Antiplatelet: |  |  |  | 0.333 |
| No | 634 (58.43%) | 550 (59.08%) | 84 (54.55%) |  |
| Yes | 451 (41.57%) | 381 (40.92%) | 70 (45.45%) |  |

Supplementary Table1. Baseline characteristics without multiple imputation for missing data.

| Variables | Threshold | Specificity | Sensitivity |
| --- | --- | --- | --- |
| Maximum.plaque.width | 0.245 | 0.5460922 | 0.5605096 |
|  |  |  |  |
| TP | 59.95 | 0.8577154 | 0.2356688 |
|  |  |  |  |
| LDL.C | 1.695 | 0.757515 | 0.3630573 |
|  |  |  |  |
| ESR | 15.5 | 0.7835671 | 0.3312102 |
|  |  |  |  |
| EO | 0.185 | 0.756513 | 0.3630573 |

Supplementary Table2. The cut-off value determined by ROC of univariate significant variables.

| Variables | Events/subjects | Univariate analysis | | | Multivariate analysis | | | |
| --- | --- | --- | --- | --- | --- | --- | --- | --- |
|  |  | OR (95% CI) | | P value | Adjusted OR (95% CI) | | P value |  |
| MACCE |  |  |  | |  |  | | |
| No Operation History | 74/653 | Reference | – | | Reference | – | | |
| Operation History | 83/345 | 2.12 (1.51-2.98) | p<0.001 | | 2.03(1.44-2.86) | P<0.001 | | |
|  |  |  |  | |  |  | | |
| TIA OR Stroke |  |  |  | |  |  | | |
| No Operation History | 93/703 | Reference | – | | Reference | – | | |
| Operation History | 64/295 | 1.64 (1.16-2.32) | p=0.005 | | 1.98(1.31-3.02) | P=0.001 | | |
|  |  |  |  | |  |  | | |
| AMI |  |  |  | |  |  | | |
| No Operation History | 19/708 | Reference | – | | Reference | – | | |
| Operation History | 19/405 | 1.73 (0.91-3.31) | p=0.097 | | 1.64(0.85-3.16) | p=0.14 | | |

Supplementary Table3. Predictive value of the operation history for primary endpoint in univariate and multivariate analysis.Model adjusted for Age, Gender, BMI, SBP, Smoke, Drunk, Diabetes, Hypertension.

| Variables | Events/subjects | Univariate analysis | | Multivariate analysis | |
| --- | --- | --- | --- | --- | --- |
|  |  | OR (95% CI) | P value | Adjusted OR (95% CI) | P value |
| MACCE |  |  |  |  |  |
| NO history of stroke | 74/653 | Reference | – | Reference | – |
| History of stroke | 83/345 | 2.12 (1.51-2.98) | p<0.001 | 1.58(1.11-2.24) | p=0.011 |
|  |  |  |  |  |  |
| TIA.or.Stroke |  |  |  |  |  |
| NO history of stroke | 47/680 | Reference | – | Reference | – |
| History of stroke | 52/376 | 2.00 (1.32-3.03) | p=0.001 | 1.92(1.25-2.93） | p=0.003 |
|  |  |  |  |  |  |
| AMI |  |  |  |  |  |
| NO history of stroke | 24/775 | Reference | – | Reference | – |
| History of stroke | 14/345 | 1.31 (0.67-2.55) | p=0.437 | 1.24 (0.62-2.48) | p=0.539 |

Supplementary Table4. Predictive value of the history of stroke for primary endpoint in univariate and multivariate analysis.Model adjusted for Age, Gender, BMI, SBP, Smoke, Drunk, Diabetes, Hypertension.

| Variables | Events/subjects | Univariate analysis | | Multivariate analysis | |
| --- | --- | --- | --- | --- | --- |
|  |  | OR (95% CI) | P value | Adjusted OR (95% CI) | P value |
| MACCE |  |  |  |  |  |
| TP<59.95 | 37/142 | Reference | – | Reference | – |
| TP>59.95 | 120/856 | 0.54 (0.36-0.81) | p=0.003 | 0.53 (0.35-0.81) | P=0.003 |
|  |  |  |  |  |  |
| TIA.OR.Stroke |  |  |  |  |  |
| TP<59.95 | 23/156 | Reference | – | Reference | – |
| TP>59.95 | 76/900 | 0.57 (0.35-0.94) | p=0.028 | 0.58 (0.36-0.98) | p=0.035 |
|  |  |  |  |  |  |
| AMI |  |  |  |  |  |
| TP<59.95 | 7/172 | Reference | – | Reference | – |
| TP>59.95 | 31/945 | 0.81 (0.35-1.86) | p=0.613 | 0.73 (0.31-1.72) | p=0.475 |

Supplementary Table5. Predictive value of the total proteins levels for primary endpoint in univariate and multivariate analysis.Model adjusted for Age, Gender, BMI, SBP, Smoke, Drunk, Diabetes, Hypertension.

| Variables | Events/subjects | Univariate analysis | | Multivariate analysis | |
| --- | --- | --- | --- | --- | --- |
|  |  | OR (95% CI) | P value | Adjusted OR (95% CI) | P value |
| MACCE |  |  |  |  |  |
| ESR<15.5 | 104/771 | Reference | – | Reference | – |
| ESR>15.5 | 53/227 | 1.73 (1.20-2.49) | p=0.003 | 1.79(1.23-2.59) | P=0.002 |
|  |  |  |  |  |  |
| TIA.OR.Stroke |  |  |  |  |  |
| ESR<15.5 | 66/809 | Reference | – | Reference | – |
| ESR>15.5 | 33/247 | 1.64 (1.05-2.55) | p=0.028 | 1.80(1.13-2.82) | P=0.011 |
|  |  |  |  |  |  |
| AMI |  |  |  |  |  |
| ESR<15.5 | 25/850 | Reference | – | Reference | – |
| ESR>15.5 | 13/267 | 1.66 (0.84-3.28) | p=0.149 | 1.56 (0.76-3.23) | P=0.227 |

Supplementary Table6. Predictive value of the ESR levels for primary endpoint in univariate and multivariate analysis.Model adjusted for Age, Gender, BMI, SBP, Smoke, Drunk, Diabetes, Hypertension.

| Variables | Events/subjects | Univariate analysis | | Multivariate analysis | | |
| --- | --- | --- | --- | --- | --- | --- |
|  |  | OR (95% CI) | P value | Adjusted OR (95% CI) | | P value |
| MACCE |  |  |  |  |  | |
| No CCBs+BBs | 93/815 | Reference | – | Reference | – | |
| CCBs+BBs | 64/140 | 4.01 (2.78-5.77) | p<0.001 | 4.06(2.72-6.07) | P<0.001 | |
|  |  |  |  |  |  | |
| TIA OR Stroke |  |  |  |  |  | |
| No CCBs+BBs | 53/855 | Reference | – | Reference | – | |
| CCBs+BBs | 46/158 | 4.70 (3.06-7.22) | p<0.001 | 4.31(2.78-6.66) | P<0.001 | |
|  |  |  |  |  |  | |
| AMI |  |  |  |  |  | |
| No CCBs+BBs | 23/855 | Reference | – | Reference | – | |
| CCBs+BBs | 15/189 | 3.05 (1.56-5.96) | p=0.001 | 2.89(1.44-5.61) | P=0.002 | |

Supplementary Table7. Predictive value of the treated with CCB combined with β-blockers for primary endpoint in univariate and multivariate analysis.Model adjusted for Age, Gender, BMI, SBP, Smoke, Drunk, Diabetes, Hypertension.
